# Cancer-Associated Fibroblasts: Their Characteristics and Their Roles in Tumor Growth

**DOI:** 10.3390/cancers7040902

**Published:** 2015-12-11

**Authors:** Kazuyoshi Shiga, Masayasu Hara, Takaya Nagasaki, Takafumi Sato, Hiroki Takahashi, Hiromitsu Takeyama

**Affiliations:** Department of Gastroenterological Surgery, Nagoya City University, 1 Kawasumi, Mizuho-cho, Mizuho-ku, Nagoya, Aichi 467-8601, Japan; kz-0926@med.nagoya-cu.ac.jp (K.S.); tnbmw532@yahoo.co.jp (T.N.); tak-sato@med.nagoya-cu.ac.jp (T.S.); takahasi@med.nagoya-cu.ac.jp (Hiroki T.); takeyama@med.nagoya-cu.ac.jp (Hiromitsu T.)

**Keywords:** cancer-associated fibroblasts, tumor-stroma interaction, origin, angiogenesis, interleukin 6, drug resistance

## Abstract

Cancer tissues are composed of cancer cells and the surrounding stromal cells (e.g., fibroblasts, vascular endothelial cells, and immune cells), in addition to the extracellular matrix. Most studies investigating carcinogenesis and the progression, invasion, metastasis, and angiogenesis of cancer have focused on alterations in cancer cells, including genetic and epigenetic changes. Recently, interactions between cancer cells and the stroma have attracted considerable attention, and increasing evidence has accumulated on this. Several researchers have gradually clarified the origins, features, and roles of cancer-associated fibroblasts (CAFs), a major component of the cancer stroma. CAFs function in a similar manner to myofibroblasts during wound healing. We previously reported the relationship between CAFs and angiogenesis. Interleukin-6 (IL-6), a multifunctional cytokine, plays a central role in regulating inflammatory and immune responses, and important roles in the progression, including proliferation, migration, and angiogenesis, of several cancers. We showed that CAFs are an important IL-6 source and that anti-IL-6 receptor antibody suppressed angiogenesis and inhibited tumor-stroma interactions. Furthermore, CAFs contribute to drug-resistance acquisition in cancer cells. The interaction between cancer cells and the stroma could be a potential target for anti-cancer therapy.

## 1. Introduction

A “tumor” is composed of cancer cells and stromal cells. The common understanding is that cancer cell is a malignant cell that does not undergo differentiation, and that stromal cells are non-malignant cells surrounding the cancer cells. Stromal cells consist of fibroblasts, vascular endothelial cells, and immune cells. Over 100 years ago, Paget *et al.* already proposed the importance of the tumor microenvironment with the theory of “seed & soil” [1]. However, for a long time, most cancer studies focused only on the “cancer cell” issue, such as alterations, including genetic and epigenetic alternation of cancer cells in carcinogenesis, progression, invasion, metastasis, and angiogenesis. Fewer studies have investigated the topic of cancer stroma as it has been thought that the stroma is just a collection of surrounding cells without a malignant function. Recently, increasing studies have clarified many aspects of the tumor-stroma interactions. Surprisingly, it was determined that stromal cells can be beneficial to the cancer cells. Thus, in order to control and eradicate cancer, we need to focus not only on the malignant cancer cells, but also the benign stromal cells.

Dvorak *et al.* proposed the theory that a “tumor is a wound that never heals” [2]. Fibroblasts in cancer tissues are similar in morphology to myofibroblasts, which are large spindle-shaped cells that are activated during the wound healing process [3]. Over 80% of stromal fibroblasts in breast cancer are thought to acquire the activated phenotype [4]. Fibroblasts, which are the major components of cancer stroma, are called cancer-associated fibroblasts (CAFs). During wound healing, when the process is completed, activated fibroblasts decrease [5]. In contrast, CAFs are perpetually activated, and neither revert to a normal phenotype nor undergoes apoptosis and elimination like normal fibroblasts [6]. To design effective therapies to target cancer, more information regarding CAFs is necessary, and novel mechanisms of CAFs are being revealed each year.

## 2. Characteristics of CAFs

### 2.1. Markers of CAFs

To detect CAFs in tumor, a specific marker is necessary. The most widely used marker for CAFs is α-smooth muscle actin (α-SMA). It has been known as a specific marker for myofibroblasts. Upon tissue damage, fibroblasts proliferate and differentiate into myofibroblasts. These myofibroblasts acquire de novo expressed α-SMA, contractile stress fibers, and the ED-A splice variant of fibronectin [5,7]. Both myofibroblasts, which are considered to be activated fibroblasts, and non-activated fibroblasts, are present in the tumor stroma. As there are more myofibroblasts in the tumor stroma, α-SMA is widely used as a CAF marker [4,8].

Another useful marker for CAFs is fibroblast activation protein (FAP), which is also a useful marker of myofibroblasts [9,10]. High intratumoral expression of FAP is associated with poor prognosis in colorectal cancer [11]. Orimo *et al.* summarized that CAFs consist of myofibroblasts and fibroblasts, and both the markers α-SMA and FAP are considered to be specific markers for myofibroblasts [8]. Nonetheless, the tissue distribution and function of FAP-α are not restricted to stromal fibroblasts: its expression is detectable in epithelial malignant cells [12,13].

Several other markers have also been reported in previous studies, such as tenascin-C [14], periostin [15], neuron glial antigen-2 (NG2) [16], vimentin, desmin, platelet derived growth factor receptor-α and β (PDGFR α and β), and fibroblast specific protein-1 (FSP-1) [17,18]. These markers are not necessarily specific for myofibroblasts. On the other hand, cytokeratin and CD31 are considered negative markers, as CAFs do not have epithelial and endothelial characteristics [19,20].

No specific marker of CAFs is known, but a combination of the above markers can help identify CAFs (Table 1).

**Table 1:** Markers of CAFs.

Positive Marker	Negative Marker
α-SMA	Cytokeratin
Fibroblast activation protein	CD31
tenascin-C	
periostin	
Neuron glial antigen-2	
Vimentin	
Desmin	
Platelet derived growth factor receptor	
Fibroblast specific protein-1	

CAFs can be isolated from various cancer types such as breast cancer, prostate cancer, pancreatic cancer, cholangiocarcinoma, lung cancer, gastric cancer, and colorectal cancer. However, CAFs are relatively rare in brain, renal, and ovarian cancers [21,22,23,24,25,26,27,28,29].

### 2.2. Heterogeneity and Origins of CAFs

**Figure 1:**
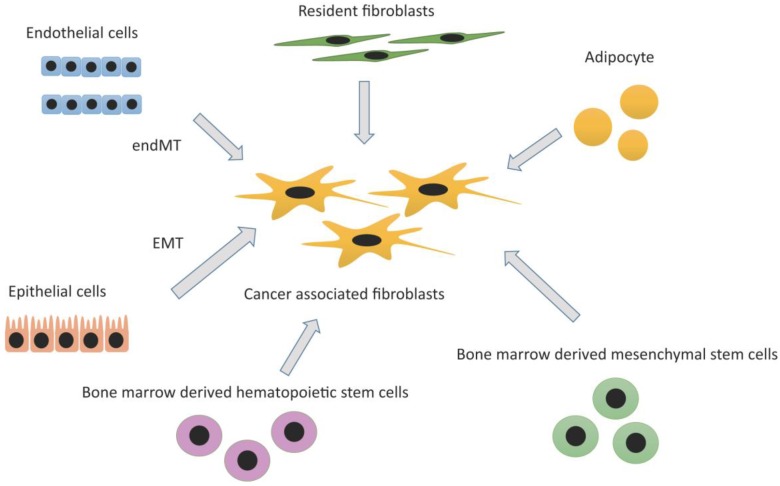
Origins of cancer-associated fibroblasts (CAFs): CAFs are considered to originate from various cells such as resident fibroblasts, adipocytes, epithelial cells (through epithelial mesenchymal transition: EMT), endothelial cells (through endothelial mesenchymal transition: endMT), bone marrow derived mesenchymal stem cells, and hematopoietic stem cells.

There are several theories regarding the origins of CAFs, and this topic is still under debate. Until now, increasing studies have reported several kinds of cells as its origins. For example, resident tissue fibroblasts, bone marrow-derived mesenchymal stem cells, hematopoietic stem cells, epithelial cells (epithelial-mesenchymal transition; EMT), and endothelial cells (endothelial-mesenchymal transition; EndMT) are all considered possible predecessors of CAFs. It is possible that CAFs are derived from several cell types, and are therefore heterogenous [8,16] (Figure 1).

#### 2.2.1. Resident Fibroblasts

A common theory of the origins of CAFs points to the resident tissue fibroblasts. Recent studies have shown that cancer cells reprogram fibroblasts to become CAFs through the actions of miRNAs (miR-31, miR-214, and miR-155) [30]. Kojima *et al.* demonstrated that resident human mammary fibroblasts can convert to CAFs during the course of tumor progression using a co-implantation tumor xenograft model [31]. Reactive oxygen species (ROS) also promote the conversion of fibroblasts into highly migrating myofibroblasts through the accumulation of hypoxia-inducible factor (HIF)-1α transcription factor and CXCL12 chemokine [32]. The recruited bone marrow derived mesenchymal stem cells (BM-MSCs) may be able to convert normal fibroblasts to CAF-like fibroblasts via alteration of secreted transforming growth factor-β1 (TGF-β1) [33]. Therefore, a plausible hypothesis is that certain cytokines secreted from cancer cells promote the conversion of resident fibroblasts into CAFs. However, CAFs and resident fibroblasts do differ in certain characteristics. Our results revealed some differences in the expression of IL-6 between CAFs derived from the malignant tumor stroma and normal stromal fibroblasts derived from the nonmalignant tumor stroma. On the other hand, the differences in the expression of IL-6 were not seen when normal stromal fibroblasts were stimulated with lipopolysaccharide (LPS) (a model of inflammation) and were cocultured with cancer cell lines. This phenomenon suggested that normal fibroblasts may convert into CAFs and acquire characteristics of CAFs [27].

#### 2.2.2. Adipocytes

Adipocytes can also be observed in cancer tissue structures. Adipose tissue-derived stem cells (ASCs) are located adjacent to cancer cells, and directly interact with tumor cells. Furthermore, similar to CAFs, adipocytes that are present in tumor stroma are called cancer associated adipocytes (CAAs), and have become another area of interest in cancer research. Using wild-type mice transplanted with GFP-labeled bone marrow, several studies have shown that CAAs are derived from circulating progenitors in the bone marrow [34,35]. CAFs may be derived at least partly from CAA [36,37]. However, there are few reports detailing the conversion from CAAs to CAFs. Jotzu *et al.* demonstrated that a significant percentage of ASCs differentiate into the α-SMA and tenascin-C positive CAF-like myofibroblastic phenotype when exposed to conditioned medium from the human breast cancer cell lines MDAMB231 and MCF7 [38].

#### 2.2.3. Bone Marrow-Derived Mesenchymal Stem Cell (MSC) and Hematopoietic Stem Cell (HSC)

There are two types of stem cells in bone marrow. One is mesenchymal stem cells (MSCs), and another is hematopoietic stem cells (HSCs). Bone marrow-derived mesenchymal stem cells differentiate into several cell types such as bone, cartilage, muscle, tendon, adipose, and stromal cells. In a study by Ishii *et al.*, the human pancreatic cancer cell line Capan-1 was subcutaneously xenotransplanted into mice that received a transplant of bone marrow cells [39]. When the tumor stroma was examined, both bone marrow-derived endothelial cells and α-SMA-positive myofibroblasts were present within and around cancer nests [39]. Similarly, using a mouse model of pancreatic insulinoma, α-SMA positive mesenchymal cells labeled with green fluorescent protein from a male donor were transplanted into a female recipient. It was reported that approximately 25% of the myofibroblasts in these pancreatic tumors are donor-derived [40]. Quante *et al.* also showed that at least 20% of CAFs originate in bone marrow and are derived from mesenchymal stem cells [41]. There are several reports regarding the mechanism of this process. Tumor-derived osteopontin (OPN) engenders MSC-to-CAF transformation in the microenvironment to promote tumor growth and metastasis via the OPN-myeloid zinc finger 1 (MZF1)-TGF-β1 pathway [42]. On the other hand, bone marrow derived hematopoietic stem cells differentiate into hematopoietic cells such as leucocytes, erythrocytes, and thrombocytes. Recent studies show that HSC can not only into these hematopoietic cells, but also CAFs [43]. In murine tumor models, a clonal population of cells derived from a single enhanced green fluorescent protein (EGFP) positive HSC was transplanted into mice. EGFP positive HSC derived cells with fibroblastic morphology and expression of type I collagen as well as α-SMA within the tumor stromal capsule were observed [43,44]. These findings suggested that bone marrow-derived MSCs and HSCs may develop into CAFs.

#### 2.2.4. Epithelial Cells: Epithelial Mesenchymal Transition (EMT)

In the early 1980s, Greenburg *et al.* proposed the term epithelial mesenchymal transition (EMT) [45]. EMT is the process by which epithelial cells with tight junctions switch to mesenchymal cells with loose cell-cell contacts and obtain mesenchymal properties. The process is observed in embryogenesis, wound healing, metastasis of cancer, and fibrosis [46,47]. Iwano *et al.* reported the origin of fibroblasts in a study using bone marrow chimeras and transgenic reporter mice. FSP1 positive fibroblasts arise in large numbers by local epithelial-mesenchymal transition (EMT) during renal fibrogenesis [48]. Petersen *et al.* showed that, under appropriate conditions, breast cancer cells may transdifferentiate to myoepithelial cells, and finally become myofibroblasts, the ancestors of CAFs [49]. However, Wang *et al.* showed that, in laryngeal xenografted tumors, CAFs are not derived from cancer cells via EMT [50], although the reason for this is not clear. Generally, it is known that EMT signals are induced mainly by TGF-β. Recent studies have shown that alveolar epithelial type 2 cell line RLE-6TN treated with TGF-β1 is converted into myofibroblasts by EMT through Ras-ERK pathway [51]. EMT is an important process in the CAFs.

#### 2.2.5. Endothelial Cells: Endothelial-Mesenchymal Transition (EndMT)

Endothelial-mesenchymal transition (EndMT) was first observed during heart formation in embryonic period [52]. During this time, TGF-β signaling switches from endothelial cells to fibroblast-like cells in the cardiac tissues [53]. Several studies showed that similar phenomenon occurs with cancer stroma. When mouse lung endothelial cells were exposed to TGF-β1, the cells acquired a spindle shaped fibroblast-like morphology. The expression of endothelial marker such as CD31 is down-regulated, while that of mesenchymal markers such as FSP-1, α SMA, and fibronectin is induced [54,55,56]. Zeisberg *et al.* demonstrated that CAFs originated from vascular endothelial cells [54].

## 3. The Role of CAFs

### 3.1. Tumor-Stroma Interaction

Decades ago, it was believed that tumor proliferation, invasion, and metastasis occur as a result of cancer progression. However, recent studies revealed that instead of cancer cells, CAFs contribute to tumor proliferation, invasion, and metastasis via secretion of various growth factors, cytokines, chemokines, and degradation of extracellular matrix (ECM) proteins [57]. A correlation of prognosis with the expression of several genes in CAFs was reported in a recent study. Strong expression of LOXL2 correlates with poor prognosis in colon cancer patients [58]. When CAFs that were isolated from a human breast carcinoma and a breast cancer cell line (MCF-7) were subcutaneously injected into immunodeficient (nude) mice, growth of the breast carcinoma with CAFs was more rapid compared to that of the breast carcinoma with normal fibroblasts because of elevated secretion of stromal-cell-derived factor 1 (SDF-1) in the former [59]. The tumor stroma, which consists of fibroblasts, ECM, vascular endothelial cells, and immune cells, acts as a barrier in promoting tumorigenesis and drug delivery. The ECM consists of fibrillar and structural proteins, proteoglycans, integrins, and proteases [60]. CAFs in rat colon carcinoma promote tumor metastasis in non-invasive cancer cells when co- injected into rats with colon carcinoma [61]. 

**Figure 2:**
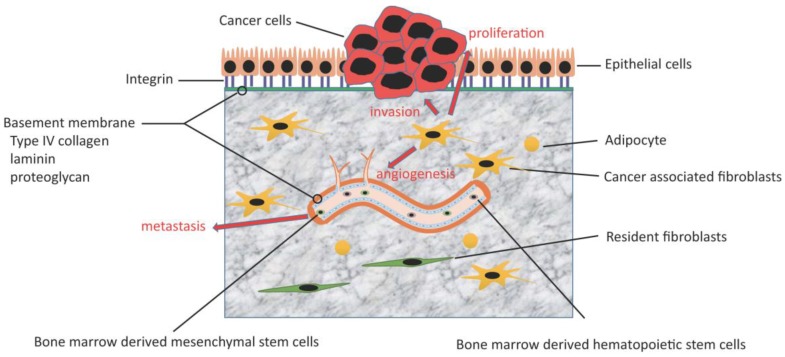
Tumor-stroma interactions and the role of CAFs. CAFs contribute to cancer proliferation, invasion, metastasis, and angiogenesis through several factors.

In order for cancer to invade and metastasize, withdrawal from the primary region is necessary. When breast cancer cells were co-cultured with normal fibroblasts, the expression of E-cadherin, an epithelial adhesion molecule, was higher than that noted for cancer cells with no fibroblasts. On the other hand, when they were cultured with CAFs, E-cadherin expression was down-regulated, as observed via immunofluorescence [62]. Furthermore, CAFs also play a role in ECM remodeling by expressing members of the matrix metalloproteinase family (MMPs). MMPs are classified as either membrane type or soluble type. MMP-2 and MMP-9 selectively degrade type IV collagen and laminin, which constitutes the basement membrane, and contribute to tumor proliferation. MMP-9 is activated by MMP-3 and MMP-13 [63]. Tumor necrosis factor-α (TNF-α) and TGF-β, which are secreted from breast cancer cells, induce the expression of MMP-9 in fibroblasts [64]. MMP-9 secretion from CAFs is suppressed by omega-3 polyunsaturated fatty acids as an anti-tumor effect [65]. As described above, CAFs contribute to cancer proliferation and invasiveness including matrix remodeling. Furthermore, CAFs modulate tumor angiogenesis. As a result, CAFs expand the tumor mass and facilitate metastasis [66] (Figure 2).

### 3.2. Angiogenesis

Angiogenesis is an essential mechanism for the development of malignant tumors. Folkman *et al.* reported that tumor growth and progression are dependent on angiogenesis, and proposed anti-angiogenic therapy as a means of treatment [67]. In 1989, Ferrara discovered vascular endothelial growth factor (VEGF), which is a key angiogenic factor [68]. The VEGF receptor was also discovered soon [69]. Based on current research, many types of cancer cells have been demonstrated to secrete VEGF themselves. These angiogenic factors are induced by hypoxic environments and various chemical factors. In response to hypoxic conditions, human mammary fibroblasts up-regulate VEGF mRNA and increase VEGF protein levels in accordance with the degree of oxygen deprivation [70]. VEGF, fibroblast growth factor (FGF), platelet-derived growth factor (PDGF), insulin-like growth factor (IGF), TGF-β, angiopoietins, and several chemokines are known as pro-angiogenic factors. Thrombospondin-1, angiostatin, and endostatin are known as anti-angiogenic factors [71]. Recent studies also showed that CAFs affect angiogenesis in the tumor by several mechanisms: via secretion of VEGF and other angiogenic factors.

#### 3.2.1. VEGF

VEGF plays a crucial role in angiogenesis and is correlated with several factors. Many investigators revealed that many kinds of cancers secrete VEGF, and the degree of its secretion influences the patient’s prognosis [72,73,74]. The roles of CAFs in tumor angiogenesis have not been clarified. Previously, our laboratory demonstrated that several types of fibroblasts release VEGF under the influence of IL-6, and the secretion levels differ among different types of fibroblasts. The levels of VEGF secretion by dermal and nonmalignant colon fibroblasts are negligible. After activation with IL-6, VEGF secretion almost reaches the level of VEGF section of CAFs. When fibroblasts that were stimulated by LPS in combination with TNF-α were used as a model of inflammation, IL-6 secretion was enhanced, and the fibroblasts acquired characteristics similar to those of CAFs. We demonstrated that normal fibroblasts secrete the same amounts of IL-6 as do CAFs and acquire characteristics of CAFs when stimulated with LPS or TNF-α [27]. We can hypothesize that some cytokine from cancer cells promotes the transition of resident fibroblasts into CAFs, and this change leads to IL-6 secretion. IL-6 is a multifunctional cytokine that plays a central role in the regulation of inflammatory and other immune responses. It has characteristics of an angiogenic cytokine. Many reports have shown that in several cancers, IL-6 performs important functions in cancer progression, including proliferation, migration, and angiogenesis [75]. We demonstrated that stromal fibroblasts isolated from colon cancer produced significant amounts of IL-6, and stimulated cancer cells into enhancing the production of IL-6 as well. Moreover, IL-6 enhanced VEGF production by fibroblasts, thereby inducing angiogenesis. *In vivo*, anti-IL6 receptor antibody targeting stromal tissue showed greater anti-tumor activity as compared to anti-IL6 receptor antibody targeting xenografted cancer cells [27].

De Boeck *et al.* examined proteins secreted by CAFs, MSCs, or recombinant TGF-β1-treated MSCs [76]. Re-analysis of these proteins by Tommelein *et al.* revealed that several proteins such as cytokines, chemokines (GDF-15, TGFβ-2, CCL-5, CXCL-12, CCL-11, CSF-1, CSF-2, IFNγ), growth factors (EGF, FGF-2, IGF-1) and other proteins (PPP2CA, PPP2R1A, CLTB, LRP-1, MMP-3, PGM-1, CD44, MFGE-8, PA2G4, UBE2D3, NRP-2, IGFBP-1, AGT, MAPK, RUVBL-1, HSPD-1) are implicated in angiogenesis [76,77].

#### 3.2.2. Other Angiogenic Factors: PDGF, FGF, and SDF-1

PDGF was isolated from platelets, and induces histogenesis during embryogenesis, is involved in wound healing, and is implicated in inflammatory diseases, ischemic cardiovascular disease, diabetes, diabetic retinopathy, and cancer. It also plays an important role in blood vessel stability. PDGF signaling regulates angiogenesis indirectly by inducing VEGF transcription and secretion directly [78]. PDGF-Rβ is predominantly expressed by tumor-associated stromal cells and pericytes of tumor vasculature in human colon carcinomas [79]. Expression of PDGF receptor on CAFs in a glycoprotein stanniocalcin-1 (STC1)-dependent manner is associated with metastasis and poor prognosis in colorectal cancer [80].

**Figure 3:**
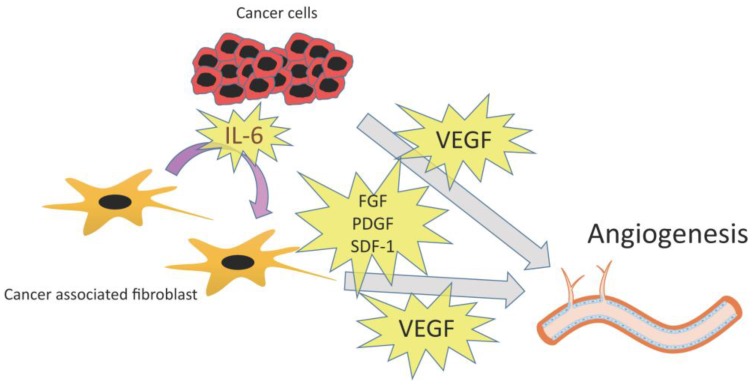
Angiogenesis and CAFs. CAFs produce IL-6. VEGF that is induced by IL-6 and several other factors (FGF, PDGF, and SDF-1) promotes angiogenesis.

FGFs have been reported to promote angiogenesis independently of VEGF [71,81]. Giulianelli *et al.* demonstrated that CAFs from hormone-independent tumors express higher levels of FGF-2 than CAFs from hormone-dependent tumors in breast cancer [82,83]. SDF-1 secreted by CAFs also contributes to angiogenesis. CAFs from breast cancer promote angiogenesis by recruiting endothelial progenitor cells through secretion of SDF-1 (CXCL-12) [59]. In summary, CAFs play a crucial role for angiogenesis through secretion of various cytokines (Figure 3).

### 3.3. Metabolism of CAFs: the Warburg Effect and Reverse Warburg Effect

In the 1920s, Otto Warburg hypothesized that, despite the presence of oxygen, most cancer cells depend on anaerobic glycolysis for ATP production, rather than on oxidative phosphorylation in the mitochondria. This phenomenon is known as “Warburg effect” [84]. In 2009, it was shown that cancer cells induce the Warburg effect in neighboring CAFs. The mechanism in cancer cells is as follows: caveolin-1 is downregulated and production of NO is increased by oxidative stress. Mitophagy and mitochondrial dysfunction are induced [85]. CAFs secrete lactate and pyruvate as energy metabolites through the glycolytic pathway [86]. Cancer cells can take up these energy metabolites and use them in the mitochondrial TCA cycle. Lisanti *et al.* called this concept the “reverse Warburg effect” [87], as CAFs contribute as the source of energy in cancer cells. Overexpression of caveolin-1 in breast cancer predicts a good outcome [88]. In recent studies, this form of energy metabolism in cancer tissues has been a target of anticancer treatments [89]. A widely studied pharmacological agent is metformin (an antidiabetic drug). Metformin activates AMPK and inhibits cancer cell proliferation through inhibition of the anaerobic glycolytic pathway [90]. There are other compounds (e.g., caffeine and rapamycin) that inhibit cancer progression via the Warburg effect and reverse Warburg effect. In breast cancer, caffeine also upregulates several proteins such as p16, p21, p53, and Cav-1 and inactivates cancer myofibroblasts [91]. Mammalian target of rapamycin (mTOR) is a known target of rapamycin. The latter drug suppresses cancer cell proliferation via inhibition of the PI3K/Akt/mTORC1 pathway [92].

### 3.4. Chemoresistance

In 1990s, Teicher *et al.* showed that breast cancer cells that are drug resistant *in vivo* no longer demonstrated significant drug resistance when they were exposed to the drugs *in vitro* [93,94]. Jain *et al.* reported that extracellular compartments such as vascular and interstitial are barriers to the delivery of therapeutic agents [93]. These reports suggested that the mechanisms of drug resistance acquisition in cancer cells are associated with not only cell autonomous processes, such as genetic and epigenetic alternations, but also with the tumor microenvironment, including CAFs. Recently, an increasing number of studies have examined the relationship between stromal cells, including CAFs, and drug resistance [95,96,97]. Meads *et al.* categorized the roles of CAFs in drug resistance, that is, in soluble factor-mediated drug resistance (SFM-DR) and cell adhesion-mediated drug resistance (CAM-DR) [98].

Straussman *et al.* investigated the effect of the tumor microenvironment on drug resistance using a co-culture system composed of 23 stromal cells and 45 cancer cell lines, with or without 35 anti-cancer drugs. Melanoma cell lines with activated BRAF proliferated and were resistant to PLX4720 (RAF inhibitor) when co-cultured with fibroblasts. Likewise, melanoma cell lines exposed to culture supernatants of the fibroblasts also demonstrated resistance. Proteomic analysis showed a correlation between stromal secretions of hepatocyte growth factor (HGF) and PLX4720 resistance [99]. When melanoma cell lines sensitive to PLX4720 were cultured in mouse subcutaneous tissues *in vitro,* they demonstrated resistance to PLX4720. However, when cultured in collagen matrix, they were not resistant to it. Furthermore, when CAFs were added to the collagen matrix, resistance to PLX4720 was observed. These results demonstrate that CAFs affect the sensitivity of drugs by secretion of various factors [100].

Recently, some studies about mechanism of the drug-resistance acquisition by CAFs has been reported. In head and neck squamous cell carcinoma, induction of MMP-1 by CAFs protected cells from cetuximab (epidermal growth factor receptor (EGFR)-targeting monoclonal antibody) [101]. The secretion of carbonic anhydrase IX (CA IX) by CAFs induced extracellular acidification and enhanced activities of MMP-2 and 9. As a result, EMT was induced and drug resistance was acquired [102]. CAFs also protected prostate cancer cells from the cytotoxic effect of sorafenib (multi-tyrosine kinase inhibitor) through over-expression of the anti-apoptotic protein BCL-XL [103]. DNA vaccine targeting the FAP, which is expressed in the tumor stroma, improved drug uptake of doxorubicin in murine colon and breast carcinoma. The combination of DNA vaccine chemotherapy is considered effective [104].

CAFs also support cancer stem cells (CSCs). CSCs are maintained in a quiescent state and are resistant to chemotherapy and radiation [105]. CSCs are selectively enriched after chemotherapy through prostaglandin E2 signaling [106]. Consequently, it is suggested that CSCs are correlated with recurrence and metastasis of cancer. In colorectal cancer, CAFs-derived conditioned medium and exosomes promoted clonogenicity and tumor growth of CSCs upon treatment with 5-fluorouracil or oxaliplatin [107]. Chemotherapy-treated CAFs maintain cancer-initiating cells (CICs) and their drug resistance through secretion of IL-17A [108]. In breast cancer, IL-6 secretion by CAFs promotes tamoxifen resistance through degradation of ER-α [109].

CAM-DR provides a therapeutic escape mediated through the adhesion of cancer cells to ECM proteins such as fibronectin, collagen, and laminin [110]. Adhesion of multiple myeloma cells to fibronectin decreased sensitivity of melphalan [111]. In ovarian cancer, they were resistant to cisplatin through over-expression of collagen VI [112]. Recent studies showed that GPER/EGFR/ERK signaling upregulates β1-integrin expression and activates downstream kinases, which contributes to fibroblast-induced cell migration and epithelial-mesenchymal transition in tamoxifen-resistant breast cancer cells. GPER most likely contributes to tamoxifen resistance via interactions with the tumor microenvironment in a β1-integrin-dependent manner [113]. Increased hyaluronan production by CAFs also induces resistance in drug-sensitive breast cancer cells [114].

Aside from tumor stroma, which acts as a structural barrier for drugs, interstitial hypertension is also a physical barrier for drug delivery [115]. Interstitial fluid pressure (IFP) is correlated with drug penetration into tumor tissue and delivery of drugs to tumor cells [110]. PDGF plays a crucial role in IFP via inhibition of the PDGF receptor, which is expressed on stromal cells, including CAFs, and decreases IFP [116,117,118]. Pharmacological agents such as imatinib reduce IFP [119].

Studies on alternative mechanisms of drug resistance related to CAFs were also reported. High mobility group box 1 (HMGB1) is a nuclear protein, and is released when cancer tissues are destroyed by chemotherapy and radiation. HMGB1 is related to drug resistance. Extracellular HMGB1 was strongly expressed in the conditioned medium after doxorubicin-induced breast cancer cell death, and its expression was higher in cells pre-treated with CAFs derived-CM than in non-tumor-associated fibroblasts-CM [120]. As illustrated above, the tumor microenvironment, especially the tumor stroma including CAFs, is crucial in cancer therapy.

## 4. Conclusions

In cancer research, “tumor microenvironment” is currently a popular topic. CAFs are a major component of tumor stroma and play a crucial role in proliferation, invasiveness, metastasis, and angiogenesis of cancer. Nonetheless, the mechanisms underlying the effects of CAFs on cancer progression are still unclear. Thus, elucidation of these mechanisms is likely to lead to new anticancer treatments targeting CAFs and the cancer-stroma interaction.

## Abbreviations

CAFs: cancer-associated fibroblasts
IL-6: interleukin-6
α-SMA: α-smooth muscle actin
FAP: fibroblast activation protein
NG2: neuron glial antigen-2
PDGFR: platelet derived growth factor receptor
FSP-1: fibroblast specific protein-1
EMT: epithelial- mesenchymal transition
EndMT: endothelial-mesenchymal transition
ROS: reactive oxygen species
HIF: hypoxia-inducible factor
BM-MSCs: bone marrow derived mesenchymal stem cells
TGFβ-1: transforming growth factor-β1
LPS: lipopolysaccharide
ASCs: adipose tissue-derived stem cells
CAAs: cancer associated adipocytes
MSC: mesenchymal stem cell
HSC: hematopoietic stem cell
OPN: osteopontin
MZF1: myeloid zinc finger 1
EGFP: enhanced green fluorescent protein
ECM: extracellular matrix
SDF-1: stromal-cell-derived factor 1
MMP: matrix metalloproteinase
TNF-α: Tumor necrosis factor-α
VEGF: vascular endothelial growth factor
FGF: fibroblast growth factor
PDGF: platelet-derived growth factor
IGF: insulin-like growth factor
STC-1: glycoprotein stanniocalcin-1
mTOR: mammalian target of rapamycin
SFM-DR: soluble factor-mediated drug resistance
CAM-DR: cell adhesion-mediated drug resistance
HGF: hepatocyte growth factor
EGFR: epidermal growth factor receptor
CSCs: cancer stem cells
CICs: cancer-initiating cells
IFP: interstitial fluid pressure
HMGB1: high mobility group box 1
